# The extent of papillary muscle approximation affects mortality and durability of mitral valve repair for ischemic mitral regurgitation

**DOI:** 10.1186/1749-8090-9-98

**Published:** 2014-06-03

**Authors:** Satoru Wakasa, Suguru Kubota, Yasushige Shingu, Tomonori Ooka, Tsuyoshi Tachibana, Yoshiro Matsui

**Affiliations:** 1 Department of Cardiovascular and Thoracic Surgery, Hokkaido University Graduate School of Medicine, Kita-15, Nishi-7, Kita-ku, Sapporo 060-8638, Japan

**Keywords:** Cardiomyopathy, Ischemic heart disease, Mitral valve, Surgery

## Abstract

**Background:**

Since reduction annuloplasty alone for ischemic mitral regurgitation (MR) cannot prevent late recurrence of MR or improve survival for those with left ventricular (LV) dysfunction, and the surgical approach to this etiology is still controversial, we conducted a study to assess the efficacy of the additional papillary muscle approximation (PMA) procedure for ischemic MR by comparing the different subtypes of PMA.

**Methods:**

We studied 45 patients who underwent mitral annuloplasty and papillary muscle approximation (PMA) for ischemic MR between 2003 and 2012. Papillary muscles were approximated entirely (cPMA: complete PMA, n = 32) through an LV incision or partially from the tips to mid-parts (iPMA: incomplete PMA, n = 13) through the mitral and aortic valves. Twenty-three patients with cPMA also underwent LV plasty (LVP). We assessed the outcomes after PMA by comparing cPMA and iPMA.

**Results:**

The baseline MR grade, NYHA class, LV end-diastolic diameter, and LV ejection fraction (LVEF) were 2.8 ± 1.0, 3.2 ± 0.6, 67 ± 6 mm, and 30 ± 10%, respectively. There were no significant differences in these parameters among those with iPMA, cPMA/LVP-, and cPMA/LVP+, though iPMA patients had better LVEF than others. Three patients died before discharge and 12 died during the follow-up. Recurrence of grade 2+ and 3+ MR occurred in 8 and 2 patients, respectively. Reoperation for recurrent MR was performed only for the 2 patients with recurrence of grade 3+ MR. The cPMA was associated with lower mortality (log-rank P = 0.020) and a lower rate of recurrence of MR ≥2+ (log-rank P = 0.005) than iPMA. In contrast, there were no significant differences in the mortality (log-rank P = 0.45) and rate of recurrence (log-rank P = 0.98) between those with cPMA/LVP- and cPMA/LVP+. The 4-year survival rate and rate of freedom from recurrence of MR ≥2+ were 83% and 85% for those with cPMA, repectively. In contrast, the rates were 48% and 48% for those with iPMA, respectively.

**Conclusions:**

Complete PMA could be associated with lower postoperative mortality and higher durability of mitral valve repair for ischemic MR.

## Background

Ischemic mitral regurgitation (MR) is a predictor of mortality in patients with ischemic heart failure [1]. This disease is predominantly caused by left ventricular (LV) remodeling and subsequent displacement of the papillary muscles [2], usually without structural valve lesions [3]. The displacement of the papillary muscles results in tethering of the mitral valve that is associated with increased stiffness of the mitral valve leaflet and loss of the normal leaflet opening and closure [4]. Since Bolling and Bach first reported the efficacy of reduction annuloplasty [5], this procedure has been the gold standard of surgical treatment for ischemic MR. However, annuloplasty alone cannot provide a survival benefit for those with LV dysfunction [6], and is associated with a high prevalence of late recurrence of MR [7], which is an important predictor of mortality after surgery for ischemic MR [8]. Reduction annuloplasty may exaggerate posterior leaflet tethering due to an anterior shift of the posterior annulus by the ring and result in recurrence of MR late after the annuloplasty [9]. Thus, the mitral valve procedure alone could be insufficient to treat ischemic MR because this is a ventricular disease rather than a valvular disease, and additional ventricular and submitral procedures could be beneficial. We adopted a submitral procedure named papillary muscle approximation (PMA) as a radical procedure for ischemic MR with severe LV dysfunction. In this procedure, we approximate the papillary muscles entirely or partially depending on the patient’s condition. We hypothesized that PMA could improve the survival and durability of mitral valve repair and the extent of approximation could affect the outcomes. We assessed the efficacy of this procedure by comparing the different subtypes of PMA.

## Methods

### Study design

Between 2003 and 2012, papillary muscle approximation was performed for 127 patients with functional MR, of whom 70 underwent this procedure for ischemic MR. Among them, 45 patients were studied after exclusion of 25 patients due to the following reasons: lack of follow-up echocardiographic data (n = 16), PMA performed as reoperation (n = 3), with a concomitant left ventricular assist device (n = 1), with another leaflet procedure (n = 1) or posterior left ventricuoplasty (n = 5). All data were collected retrospectively from medical records and our database. The study protocol was approved by the Institutional Review Board of the Hokkaido University Hospital for Clinical Research.

### Examinations of cardiac parameters

Echocardiography was performed within 1 week before surgery and repeated before discharge at 0.9 ± 0.7 months (range, 0.2 to 4.2 months) after surgery. After discharge, follow-up echocardiography was performed regularly on an outpatient basis. The latest echocardiographic study was performed at a mean follow-up time of 2.6 ± 2.2 years. The following cardiac parameters were acquired: LV end-diastolic diameter, LV end-systolic diameter, LV ejection fraction (LVEF), MR grade, deceleration time, coaptation height of the mitral valve, and interpapillary muscle distance. The LV end-diastolic and end-systolic diameters were measured by M-mode transthoracic echocardiography. The LVEF was calculated by the modified Simpson method. The severity of MR was graded based on color Doppler images as follows: 1+, mild; 2+, moderate; 3+, moderately severe; and 4+, severe [10]. The deceleration time of the early transmitral flow velocity was measured on an apical long-axis color Doppler flow image. The coaptation height of the mitral valve was measured in 2- and 4-chamber apical views in mid-systole by B-mode transthoracic echocardiography [11]. The interpapillary muscle distance was also measured in the short axis view at the papillary muscle level in end-diastole [12]. Mitral valve tethering was defined as significant if the coaptation height of the mitral valve was ≥10 mm or interpapillary muscle distance was ≥30 mm. The LV end-systolic volume was evaluated in 40 (89%) patients using echocardiography (N = 12, 30%), quantitative gated scintigraphy (N = 17, 43%), magnetic resonance imaging (N = 2, 5%) and left ventriculography (N = 9, 22%).

### Operative procedures

**Figure 1:**
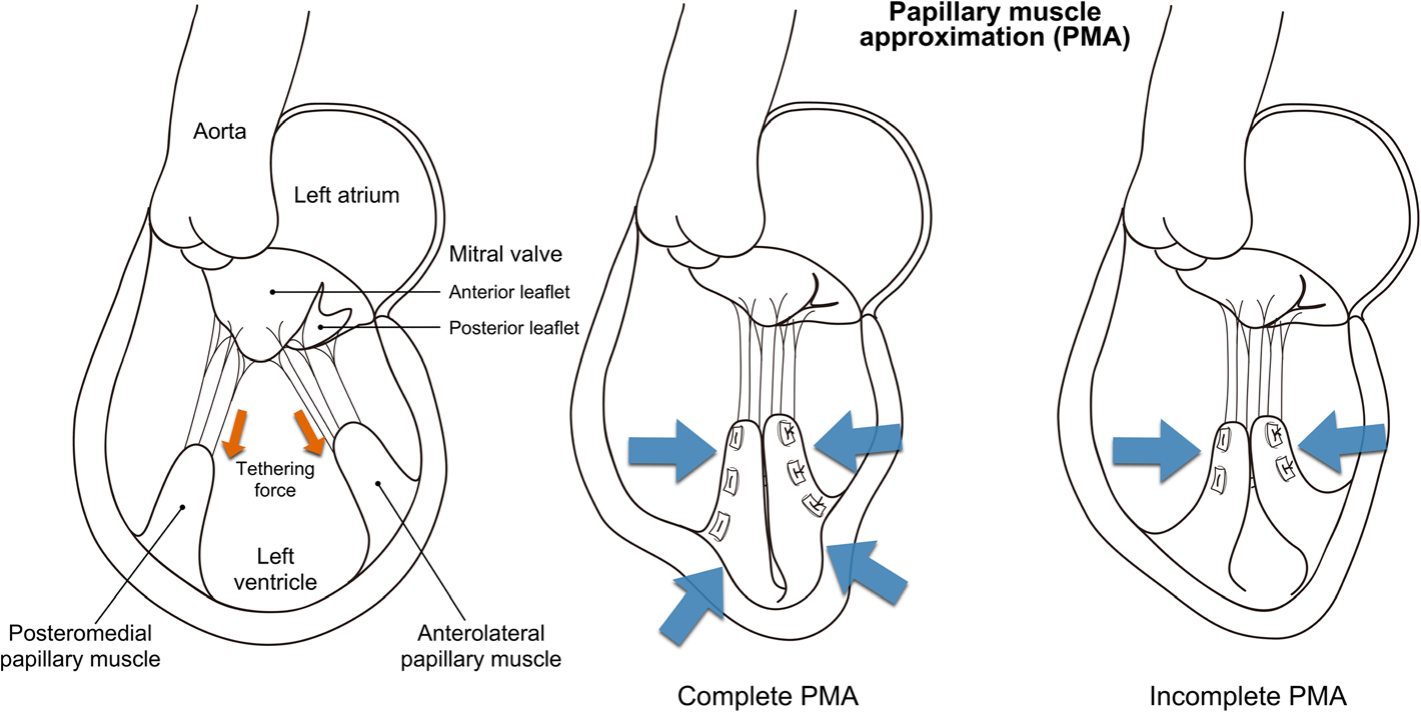
Schematic of complete and incomplete papillary muscle approximation procedures.

All the procedures were performed through a median sternotomy. Cardiopulmonary bypass was commenced through the ascending aorta and both vena cavae. After aortic cross clamping, blood cardioplegia was administrated and cardiac arrest was obtained. Subsequent procedures were performed under moderate hypothermia. Mitral annuloplasty was performed for all the patients who were indicated for PMA. We used a true-sized semi-rigid (Physioring or Physioring II, Edwards Lifesciences, Irvine, CA, USA) or rigid (Rigid Saddle Annuloplasty Ring, St. Jude Medical, Inc., St Paul, MN, USA) annuloplasty ring. The patients with significant mitral valve tethering underwent PMA, which we adopted to correct tethering by gathering the papillary muscles that were displaced due to LV remodeling. The details of the procedure were described elsewhere [13]. The papillary muscles were entirely approximated side-by-side (complete PMA) through an LV incision or partially from the tips to the mid-parts (incomplete PMA) through the mitral or aortic valve using pledgetted mattress sutures of 3–0 polypropylene (Figure 1). The method was selected based on the presence of a myocardial scar lesion on the anterior LV wall. If one existed, an LV incision was made on the anterior LV wall and complete PMA was performed. Otherwise, incomplete PMA without an LV incision was performed because entire approximation through the cardiac valves is technically difficult, especially at the bases of papillary muscles. The presence of a myocardial scar was evaluated preoperatively using quantitative gated scintigraphy, positron emission tomography, or magnetic resonance imaging. The LV was closed after complete PMA with direct suturing or concomitant left ventriculoplasty (LVP), if indicated.

The patients with severe LV dilatation (end-diastolic diameter ≥65 mm) and a broad anterior myocardial scar underwent overlapping left ventriculoplasty, which was developed as a radical procedure to reduce the LV volume and restore the LV shape and function. The details of the procedure were described elsewhere [14]. Papillary muscle suspension was adopted from 2005 to reinforce the effect of PMA by suspending the approximated tips of the papillary muscles to the mitral annulus using 4–0 polytetrafluoroethylene sutures. Initially, the suspension was performed toward the posterior mitral annulus against apical tethering, though anterior suspension became predominant from 2008, aiming at reduction of posterior tethering. Coronary artery bypass grafting was performed if the patient had untreated and significant coronary lesions. The Maze procedure and tricuspid annuloplasty were performed when needed. Tricuspid annuloplasty was indicated even for mild regurgitation if the patients had severely deteriorated LV function or pulmonary hypertension. Perioperative hemodynamic instability refractory to medical control was treated with percutaneous cardiopulmonary support and intraaortic balloon pumping.

### Statistical analyses

Continuous variables are expressed as mean ± standard deviation and categorical variables as numbers and percentages. Preoperative and postoperative data were compared using the Wilcoxon signed-rank test. Comparisons among 3 groups were conducted using the Kruskal-Wallis test for continuous data. Post hoc tests were performed using the Mann–Whitney U test with Bonferroni’s correction. Categorical data were compared using the chi-square test or Fisher’s exact test, if appropriate. Postoperative mortality and freedom from recurrence of MR were estimated using the Kaplan–Meier method, and differences among groups were assessed by the log-rank test. A two-sided P value <0.05 was considered to indicate statistical significance in all the tests. All analyses were performed using IBM SPSS Statistics (version 20, IBM Corporation, Somers, NY, USA).

## Results

### Patients’ baseline characteristics

Table 1 shows the patients’ baseline characteristics. The mean age of the study subjects was 62 ± 12 (range, 32 to 85) years and 38 (84%) were male. The baseline New York Heart Association (NYHA) functional class was III/IV for 39 (87%) patients. Complete PMA without LVP was associated with younger age. Coronary artery lesions and the severity of heart failure did not significantly differ among the 3 groups.

**Table 1:** Patients’ baseline characteristics

	**Incomplete PMA**	**Complete PMA**		**P Value**
	**(N = 13)**	**LVP-(N = 9)**	**LVP + (N = 23)**	
Age, years	65 ± 8	50 ± 14	65 ± 10	0.024
Male, n (%)	11 (85%)	9 (100%)	18 (78%)	0.48
Hypertension, n (%)	8 (62%)	4 (44%)	7 (30%)	0.19
Dialysis, n (%)	2 (15%)	2 (22%)	2 (9%)	0.62
Diabetes, n (%)	7 (54%)	4 (44%)	10 (44%)	0.92
Atrial fibrillation, n (%)	2 (15%)	0	4 (17%)	0.53
History of VT, n (%)	0	1 (11%)	5 (22%)	0.21
Coronary lesions, n (%)				
Left anterior descending	12 (92%)	8 (89%)	23 (100%)	0.23
Left circumflex	12 (92%)	8 (89%)	16 (70%)	0.26
Right	13 (100%)	7 (78%)	18 (78%)	0.13
NYHA class, n (%)				0.43
II	3 (23%)	2 (22%)	1 (4%)	
III	6 (46%)	5 (56%)	14 (61%)	
IV	4 (31%)	2 (22%)	8 (35%)	
Inotrope, n (%)	2 (15%)	1 (11%)	3 (13%)	1.0
Urgent, n (%)	2 (15%)	0	2 (9%)	0.65
IABP, n (%)	2 (15%)	0	6 (26%)	0.26
PCPS, n (%)	0	0	1 (4%)	1.0

PMA = papillary muscle approximation, LVP = left ventriculoplasty, VT = ventricular tachyarrhythmia, NYHA = New York Heart Association, IABP = intraaortic balloon pumping, PCPS = percutaneous cardiopulmonary support.

### Surgical procedures

Table 2 summarizes the operative procedures. Mitral annuloplasty was perforemed for all the patients. Complete and incomplete PMA were performed for 32 (71%) and 13 (29%) patients, respectively. Twenty-three (72%) of those with complete PMA underwent concomitant LVP. All the patients with LVP underwent overlappling left ventriculoplasty. Papillary muscle suspension was performed for 24 (53%) patients. Anterior and posterior suspension were performed for 14 and 8 patients, respectively. Coronary artery bypass was performed for 41 (91%) patients.

**Table 2:** Operative procedures

	**Incomplete PMA**	**Complete PMA**		**P Value**
	**(N = 13)**	**LVP-(N = 9)**	**LVP + (N = 23)**	
Mitral annuloplasty, n (%)	13 (100%)	9 (100%)	23 (100%)	N/A
Ring size, n (%)				0.30
24 mm	0	0	1 (4%)	
26 mm	6 (46%)	6 (67%)	17 (74%)	
28 mm	6 (46%)	3 (33%)	3 (13%)	
30 mm	1 (8%)	0	2 (9%)	
PMA, n (%)				<0.001
Complete PMA	0	9 (100%)	23 (100%)	
Incomplete PMA	13 (100%)	0	0	
PM suspension, n (%)				0.29
None	5 (39%)	3 (33%)	15 (65%)	
Anterior	6 (46%)	3 (33%)	5 (22%)	
Posterior	2 (15%)	3 (33%)	3 (13%)	
Overlapping left ventriculoplasty, n (%)	0	0	23 (100%)	<0.001
CABG, n (%)	13 (100%)	8 (89%)	20 (87%)	0.41
TAP, n (%)	9 (75%)	4 (44%)	17 (74%)	0.34
Maze, n (%)	1 (8%)	0	2 (9%)	1.0

PMA = papillary muscle approximation, LVP = left ventriculoplasty, PM = papillary muscles, CABG = coronary artery bypass grafting, AVR = aortic valve replacement, TAP = tricuspid annuloplasty, AVR = aortic valve replacement.

### Cardiac sizs and function

**Figure 2:**
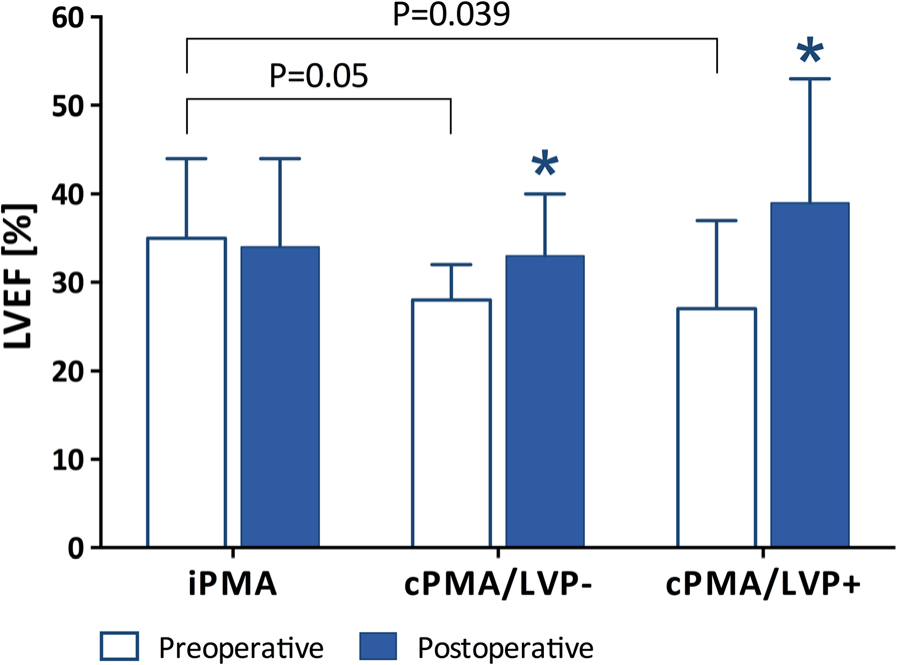
**Comparison of pre- and postoperative LVEF among those with iPMA, cPMA/LVP-, and cPMA/LVP+.** cPMA = complete papillary muscle approximation, iPMA = incomplete PMA, LVEF = left ventricular ejection fraction, LVP = left ventriculoplasty. *P < 0.05 compared with preoperative value.

Table 3 summarizes the perioperative cardiac sizes and functions. The baseline LV size, MR grade, and severity of mitral valve tethering did not significantly differ among the 3 groups. However, incomplete PMA was associated with better baseline LVEF. Postoperatively, the LV size, MR grade, coaptation height, and interpapillary muscle distance decreased significantly for all 3 groups. The LVEF significantly increased for those with complete PMA, though there was no significant change in LVEF for those with incomplete PMA (Figure 2). These postoperative parameters did not significantly differ among the 3 groups.

**Table 3:** Perioperative cardiac sizes and functions

	**Incomplete PMA (A)**	**Complete PMA/LVP- (B)**	**Complete PMA/LVP+ (C)**		**P values†**	
				**A vs. B**	**A vs. C**	**B vs. C**
Preoperative values						
LVDd, mm	66±5	65±5	68±7	1.0	1.0	0.64
LVDs, mm	55±7	57±6	58±8	1.0	0.42	1.0
LVESVI, ml/m^2^	95±24	112±26	117±46	0.74	0.50	1.0
LVEF, %	35±9	28±4	27±10	0.05	0.039	0.84
MR grade	2.8±1.0	2.9±1.1	2.8±1.1	1.0	1.0	1.0
DCT, ms	208±71	159±47	151±44	0.39	0.09	1.0
IPMD, mm	31±7	28±4	29±5	0.71	1.0	1.0
CH (2CV), mm	11±2	9±2	10±2	0.26	0.24	1.0
CH (4CV), mm	9±2	10±3	10±2	1.0	1.0	1.0
Postoperative values						
LVDd, mm	57±6**	58±4**	63±8**	1.0	0.06	0.23
LVDs, mm	48±7**	48±6**	51±11**	1.0	0.35	0.61
LVESVI, ml/m^2^	64±14**	78±25*	70±26**	0.90	1.0	1.0
LVEF, %	34±10	33±7*	39±14**	1.0	0.78	0.85
MR grade	0.4±0.5**	0.3±0.4**	0.4±0.7**	1.0	1.0	1.0
DCT, ms	274±61*	205±70	207±60*	0.25	0.06	1.0
IPMD, mm	10±7**	7±7*	5±6**	1.0	0.32	1.0
CH (2CV), mm	5±3*	4±3*	3±3**	1.0	1.0	1.0
CH (4CV), mm	4±3**	4±2*	4±3**	1.0	1.0	1.0

PMA = papillary muscle approximation, LVP = left ventriculoplasty, LV = left ventricle, LVDd = LV end-diastolic diameter, LVEF = LV ejection fraction, LVESVI = LV end-systolic volume index, MR = mitral regurgitation, DCT = deceleration time, IPMD = interpapillary muscle distance, CH = coaptation height of the mitral valve, 2CV = 2-chamber apical view, 4CV = 4-chamber apical view.

†Bonferroni corrected P.

*P <0.05 or **P<0.01 compared with preoperative values.

### Postoperative mortality

**Figure 3:**
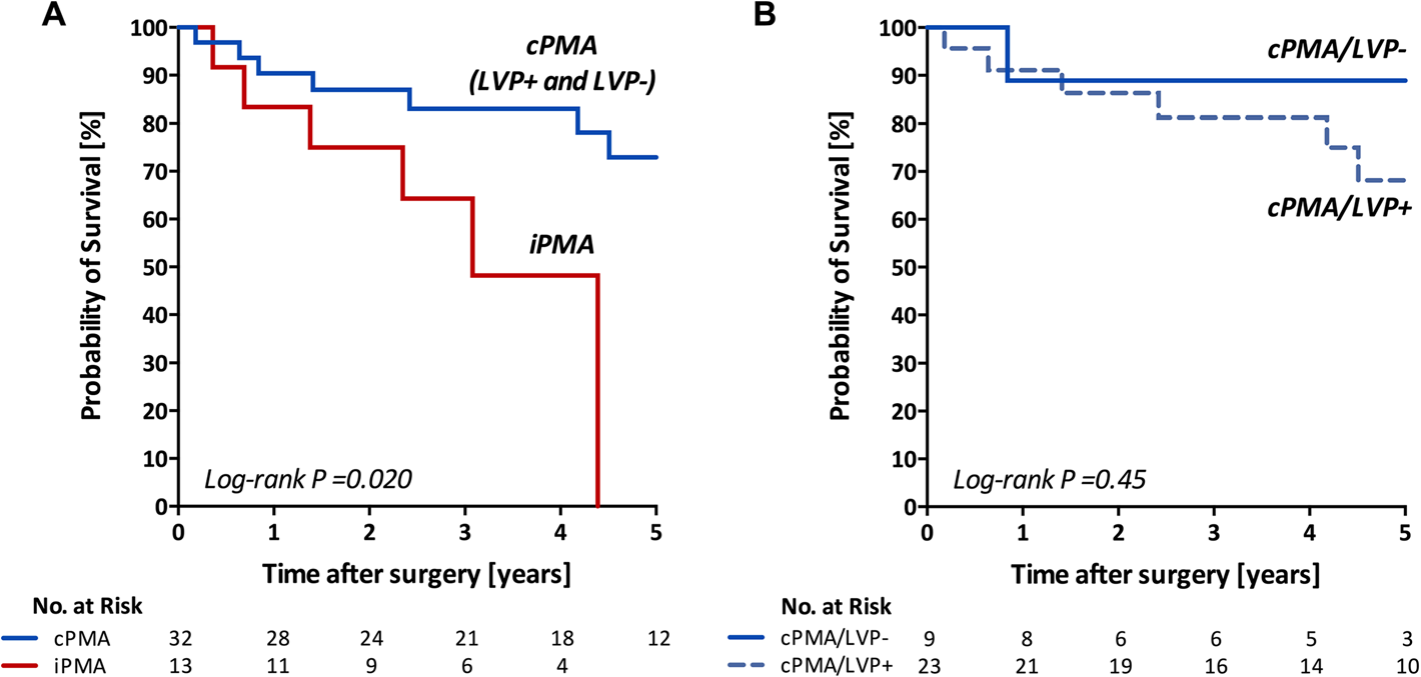
**Comparison of survival between complete and incomplete papillary muscle approximation procedures (A), and complete papillary muscle approximation with and without left ventriculoplasty (B).** cPMA = complete papillary muscle approximation, iPMA = incomplete PMA, LVP = left ventriculoplasty.

Three patients died before discharge and 12 died during a mean follow-up time of 3.5 ± 2.3 years. Cardiac-related deaths were observed for 3, 1, and 4 patients, respectively (P = 0.78). Other non-cardiac causes of death were septicemia for 4, bowel ischemia for 1, stroke for 1, and suicide for 1. Hospital mortality rates were 15%, 0%, and 4.3% for those with incomplete PMA, complete PMA/LVP-, and complete PMA/LVP+, respectively (P = 0.42). The postoperative mortality was significantly different between those with complete and incomplete PMA (log-rank P = 0.020, Figure 3A). The 1-, 2-, and 4-year survival rates were 90%, 87%, and 83% for those with complete PMA, respectively. In contrast, those rates were 83%, 75%, and 48% for those with incomplete PMA, respectively. Concomitant LVP and papillary muscle suspension did not affect mortality. There was no significant difference in mortality between those with complete PMA/LVP + and complete PMA/LVP- (log-rank P = 0.45, Figure 3B) or among those with anterior papillary muscle suspension, posterior suspension, and without suspension (log-rank P = 0.48).

The postoperative NYHA functional class was class II or less in all but 3 patients with class III and did not significantly differ among the 3 groups (P = 0.76). At the latest follow-up, most (88%) of the patients had NYHA functional class II or less.

### Recurrence of MR

**Figure 4:**
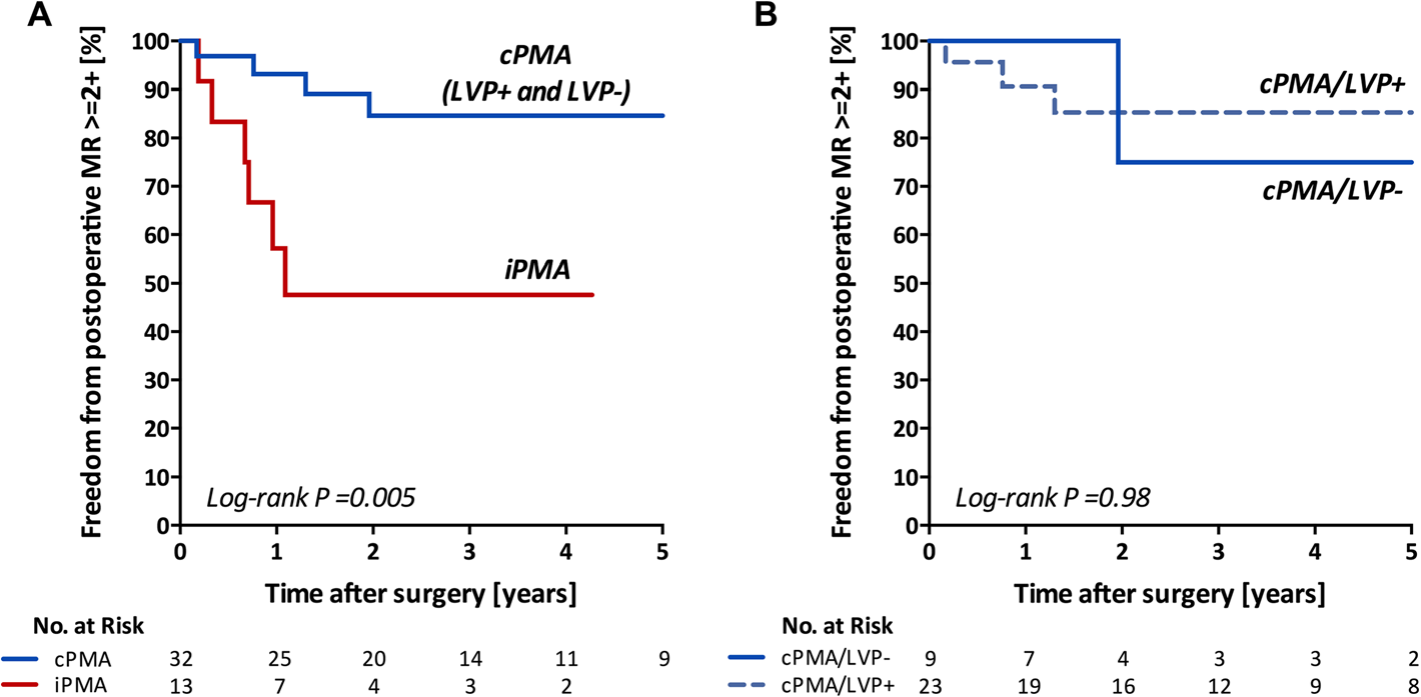
**Comparison of freedom from recurrence between complete and incomplete papillary muscle approximation procedures (A) and complete papillary muscle approximation with and without left ventriculoplasty (B).** cPMA = complete papillary muscle approximation, iPMA = incomplete PMA, LVP = left ventriculoplasty.

Recurrence of MR ≥2+ was observed in 10 patients, of whom only 2 had grade 3+. No patient had grade 4+ recurrence. Reoperation for recurrent MR was performed for the 2 patients with recurrence of grade 3+ MR. One had incomplete PMA and presented recurrent MR due to infective endocarditis and underwent mitral valve replacement with redo PMA 2 months after the initial operation. The other had early postoperative recurrence of MR after complete PMA due to dehiscence of the PMA sutures. He underwent redo PMA but died before discharge due to mediastinitis 17 months after the initial operation. Kaplan-Meier curves for the rate of recurrence of MR significantly differed between those with complete and incomplete PMA (log-rank P = 0.005, Figure 4A). The 1-, 2-, and 4-year rates of freedom from recurrence were 93%, 85%, and 85% for those with complete PMA, respectively. In contrast, those rates were 57%, 48%, and 48% for those with incomplete PMA, respectively. Concomitant LVP and papillary muscle suspension did not affect the durability of the mitral valve repair. The rates of freedom from recurrence did not significantly differ between those with complete PMA/LVP- and complete PMA/LVP + (log-rank P = 0.98, Figure 4B) or among those with different types of papillary muscle suspension (log-rank P = 0.43).

## Discussion

In this study, we assessed the results of PMA for ischemic MR and demonstrated that mitral valve repair with complete PMA was associated with a satisfactorily high postoperative survival rate and durability of repair considering the results of reduction annuloplasty alone in previous reports. In addition, the extent of approximation affected the outcomes. The approximation of the entire papillary muscles was associated with better outcomes than that partially from the tips to mid-parts of the papillary muscles.

The indication for mitral valve repair of ischemic MR still remains controversial because its benefit has not been proven. Wu et al. reported that mitral annuloplasty provided no significant survival benefit for those with moderate to severe MR and LV dysfunction [6]. In contrast, a recent subanalysis of the STICH trial showed that adding mitral valve repair to CABG could be beneficial for those with LV dysfunction and moderate to severe MR compared with CABG alone or medical treatment alone [15]. However, even with mitral valve repair, postoperative mortality is still high in this clinical setting. The 5-year mortality rates were 41%, 54%, and 55% for those with mitral valve repair with CABG, CABG alone, and medication alone, respectively. Mitral valve repair with reduction annuloplasty alone for ischemic MR frequently results in late recurrence of MR. The reported rates of recurrence are between 15% and 25% within 6 months and approximately 70% at 5 years [7]. The issue of durability of the repair may be critical because recurrence of MR is associated with mortality [8]. However, it remains a matter of debate whether mitral valve replacement rather than repair should be selected to prevent recurrence. A recent study demonstrated that there was no significant difference in postoperative survival between mitral valve repair and replacement, though reoperation is frequently associated with repair due to recurrence [16]. In contrast, De Bonis et al. reported that mitral valve repair was preferable to replacement in terms of postoperative survival and improvement of cardiac functions [17]. Thus, reduction annuloplasty alone is not always a suitable surgical option for ischemic MR in terms of the mortality and recurrence of MR, though mitral valve replacement cannot provide a further benefit. The severity of LV remodeling may have a great impact on the results because ischemic MR is a ventricular disease rather than a simple valvular disease. Indeed, a dilated LV (≥65 mm) is associated with poor outcomes after reduction annuloplasty for ischemic MR [18]. Therefore, an additional procedure such as a ventricular or submitral one, could be required for improvement of the outcomes.

We have adopted papillary muscle approximation to correct mitral valve tethering for functional MR since 2003 [13]. In this procedure, the outward tethering is directly corrected by gathering the papillary muscles together. Apical and posterior tethering can also be improved because the LV dimension decreases due to inward traction of the LV wall by the approximated papillary muscles. Those corrections of tethering may result in a reduction of leaflet tension and restoration of normal leaflet motion. Therefore, we adapt a true-sized annuloplasty ring for mitral valve repair with PMA because a downsized annuloplasty ring may not be necessary after the correction of mitral valve tethering. The use of a true-sized ring might also prevent functional mitral stenosis that could occur after reduction annuloplasty [19].

There are two different types of PMA in terms of the extent of approximation: complete and incomplete PMA. Although procedures similar to both complete [20,21] and incomplete [22] PMA have been proposed, the appropriate concept for this procedure remains unestablished. In this study, we demonstrated that complete PMA was associated with lower mortality and higher durability of repair than incomplete PMA. This is consistent with the report by Jensen et al. They proposed that the traction of the basal papillary muscle was preferable to traction of the papillary muscle tip because instability of the LV wall attached by the papillary muscles might permit LV remodeling and result in recurrence of MR [23]. The inferoposterior LV wall function could play an important role in the treatment of functional MR. Regional remodeling including posteromedial papillary muscles after inferior myocardial infarction causes ischemic MR with a smaller degree of LV dilatation and dysfunction than anterior infarction [24]. In addition, Pocar et al. reported that impaired regional inferoposterior wall motion was a predictor of the recurrence of MR after reduction annulopalsty in patients with ischemic MR [25]. Complete PMA causes regional surgical reverse remodeling at the inferoposterior LV wall and stabilizes the LV wall at the base of the papillary muscles as after papillary muscle imbrication [26]. This could explain why complete PMA was associated with significant improvement of LVEF after surgery but incomplete PMA was not. All the patients enrolled into our study would have had significant regional remodeling in the inferoposterior wall with a great interpapillary muscle distance. Therefore, complete PMA should be considered even with a transvalvular approach to the degree possible. Otherwise, alternative procedures with new approaches, such as extracardiac and interventional approaches, to the basal papillary muscles will be required for the improvement of the results.

Some concomitant procedures such as LVP could bias the results of PMA because LVP causes radical changes of LV geometry and functions, resulting in surgical reverse remodeling of the LV. Although we found no significant difference in mortality and the durability of repair between those with complete PMA/LVP + and those with complete PMA/LVP-, there was an inherent difference in ventricular scar size, which could affect the outcomes. Thus, left ventriculoplasty might improve the results of those with large ventricular scars and diminish the possible difference in outcomes between those with complete PMA/LVP + and complete PMA/LVP-. However, there was still a significant difference in mortality between those with incomplete PMA and those with complete PMA/LVP- (log-rank P = 0.047). Therefore, concomitant left ventriculoplasty to some extent influenced the outcomes of complete PMA cases but did not significantly affect our conclusions. In contrast, papillary muscle suspension could modify the effect of PMA. Especially with anterior suspension, further improvement of posterior tethering could be expected. In our study, however, papillary muscle suspension did not demonstrate a significant effect on survival or the durability of repair.

### Limitations

There were several limitations to this study that should be mentioned. First, the small sample size with a variety of surgical procedures might have affected the statistical significance. Because of the long study period with respect to the sample size, a learning curve might affect the outcomes. However, there was no significant difference in mortality between those who underwent surgery in the early (before 2006, N = 18) and late (after 2007, N = 27) eras (log-rank P = 0.76). The rate of freedom from recurrence ≥2+ was also comparable between the groups (log-rank P = 0.11). Second, we did not compare PMA and annuloplasty alone because the baseline characteristics and cardiac parameters in both populations were significantly different in our cohort. Instead, we evaluated the outcomes after PMA compared to the results of annuloplasty alone in previous reports. A future study comparing PMA and annuloplasty with a larger sample size will help further our understanding.

## Conclusions

Complete PMA appears to be associated with a high survival rate and the durability of mitral valve repair for ischemic MR with severe LV remodeling. Approximation of the basal papillary muscles with the surrounding LV wall could be an important concept for the repair of ischemic MR.

## Abbreviations

LV: Left ventricle; LVEF: Left ventricular ejection fraction; LVP: Left ventriculoplasty; MR: Mitral regurgitation; NYHA: New York Heart Association; PMA: Papillary muscle approximation.

## Competing interests

The authors declare that they have no competing interests.

## Authors’ contributions

SW conceived and designed the study, collected the patients’ data and drafted the manuscript. SK participated in the design of the study and helped to collect the patients’ data. YS helped to design the study, draft the manuscript and perform statistical analysis. OT and TT participated in the design of the study and helped to draft the manuscript. YM developed the surgical techniques and helped to draft the manuscript. All authors read and approved the final manuscript.
